# Supplementary material for: PVP1—The People’s Ventilator Project: A fully open, low-cost, pressure-controlled ventilator research platform compatible with adult and pediatric uses
Source: PLoS One. 2022 May 11;17(5):e0266810. doi: 10.1371/journal.pone.0266810 (PMC9094548; doi:10.1371/journal.pone.0266810)
Supplement: S1 Appendix — The appendix contains the complete set of EUA ISO standard tests [22], elaborates on the design, and provides calibration and validation data. (PDF) [file pone.0266810.s001.pdf]

Appendix: Further tests, and validation data of PVP1

Introduction

This supplemental material serves three purposes:

- Show the complete set of EUA ISO Standard Tests for an adult lung.
- Make explicit further design considerations that went into hardware and software design.
- Enumerate and describe, in detail, the assembly, and function of all parts.
- Provide extensive calibration and validation data, specifically focusing on the compromises made with our selection of parts.

For further details, we also refer the reader to our online reponsitories, that contain all code, and detailed build instructions:

- Source Code and Community
- Documentation
- PyPi - `pip install pvp`

Contents

1 Validation Tests and EUA ISO Standards **2**

2 Hardware Design **2**
- 2.1 Overview . . . 2
- 2.2 Actuator Selection . . . 7
- 2.3 Sensor Selection . . . 8
- 2.4 Electronics Design . . . 9

3 Software Design **11**
- 3.1 Software Architecture . . . 11
- 3.2 Supported Alarms . . . 15
- 3.3 Alarm Design . . . 15

4 Calibration and System Limitations **19**
- 4.1 Calibration of the Flow Sensor . . . 19
- 4.2 Hardware Delay Characterization . . . 20

1 Validation Tests and EUA ISO Standards

EUA ISO Standard Tests

The FDA EUA guidelines lay out standardized tests required for all pressure controlled ventilation (see ISO 80601-2-80:2018). We performed this battery of tests (with the exception of those requiring controlled leak rates) and present the results in Figs 1 and 2. These tests cover an array of conditions, and more difficult test cases involve a high airway pressure coupled with a low lung compliance (case nos. 8 and 9 in Fig 2). Under these conditions, if the inspiratory flow rate during the ramp phase is too high, the high airway resistance will produce a transient spike in airway pressure which can greatly overshoot the PIP value. For this reason, the system uses a low initial flow setting and allows the clinican to increase the flow rate if necessary (cf. Fig 3A in the main manuscript).

The PVP1 integrates expiratory flow to monitor the tidal volume, which is not directly set in pressure controlled ventilation, but is an important parameter to ensure sufficient oxygen is delivered to the lungs. Of the test conditions in the ISO standard, four that we tested intended a nominal delivered tidal volume of 500 mL, three intended 300 mL, and one intended 200 mL. For most cases, the estimated tidal volume has a tight spread clustered within 20% of the intended value (see Fig 3), consistent with the performance in a pediatric setting (cf. Fig 4 in the main manuscript).

The choice of items to display on the graphical user interface (GUI) was done in close interaction with clinicians. Specifically, we chose to display VTE (the end-tidal volume; the amount of air the patient's lungs return to the ventilator with exhalation), mean airway pressure (MAP), peak inspiratory pressure (PIP), and positive end-expiratory pressure (PEEP), but the modular design of the GUI allows users to easily configure a different set of display and control values.

2 Hardware Design

2.1 Overview

The device components were selected to enable a minimalistic and relatively low-cost ventilator design, to avoid supply chain limitations, and to facilitate rapid and easy assembly. Most parts in our system are not medical-specific devices, and those that are specialized components are readily available and standardized in the United States across ventilator platforms, such as respiratory circuits and HEPA filters. We are providing a complete assembly of the device, including 3D-printable components, as well as justifications for selecting all actuators and sensors in the sections below, as guidance to those who cannot source an exact match to components used here. Readers may refer to the system schematic in Fig 1 in the main manuscript, and the schematic in Fig 4A here.

Hospital Gas Blender

At the inlet to the system, we assume the presence of a commercial-off-the-shelf (COTS) gas blender. These devices mix air from U.S. standard medical air and O2 as supplied at the hospital wall at a pressure of around 50 psig. The device outlet fitting may vary, but we assume a male O2 DISS fitting (NIST standard). In field hospitals, compressed air and O2 cylinders may be utilized in conjunction with a gas blender, or a low-cost Venturi-based gas blender. We additionally assume that the oxygen concentration of gas supplied by the blender can be manually adjusted. Users will be able to monitor the oxygen concentration level in real-time on the device GUI.

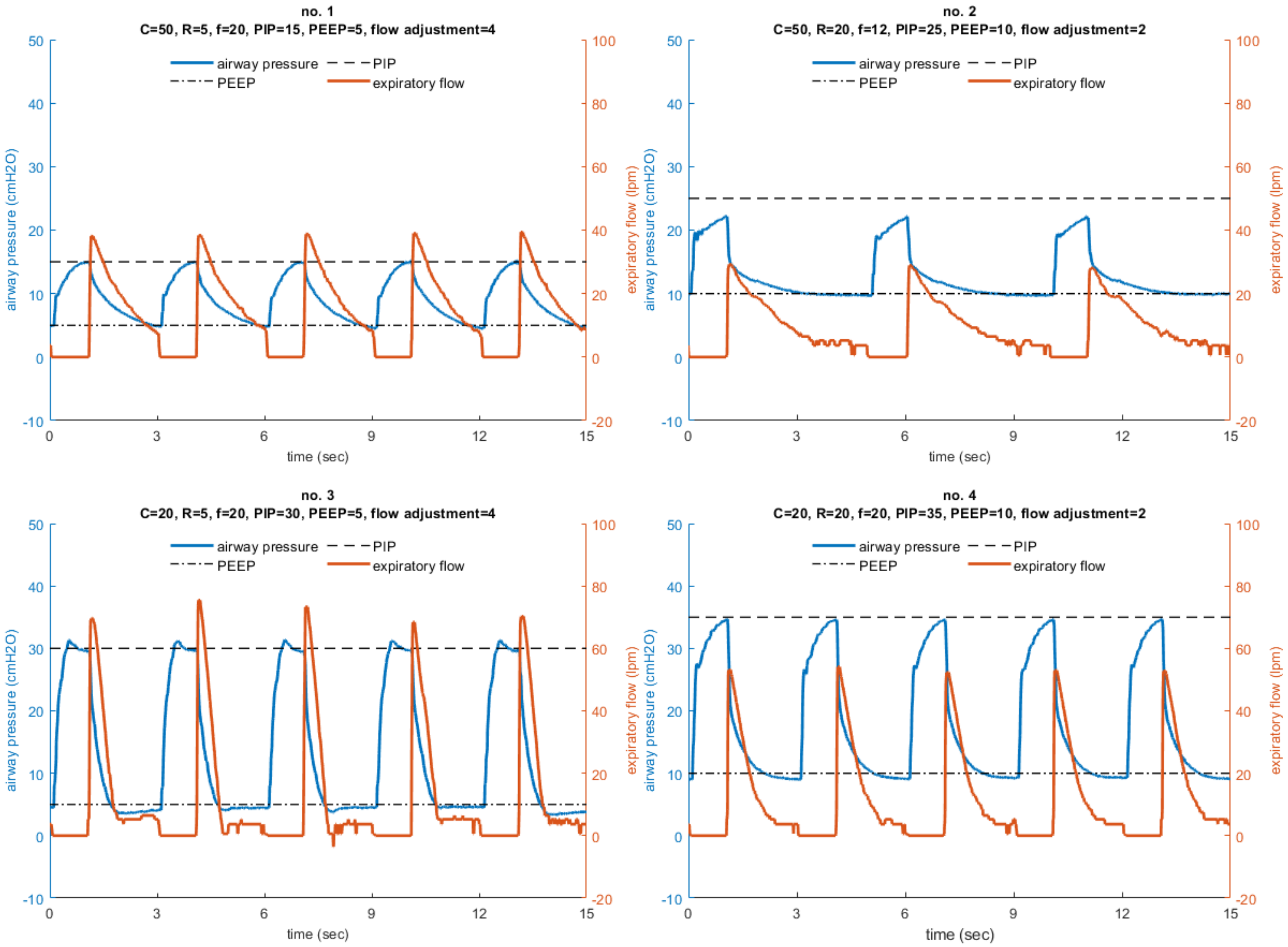

Fig 1. Performance results of the ISO 80601-2-80:2018 pressure controlled ventilator standard tests with an intended delivered tidal volume of 500 mL. For each configuration the following parameters are listed: the test number (from table 201.105 in the ISO standard), the compliance (C, mL/cmH_2O), linear resistance (R, cmH_2O/L/s), respiratory frequency (f, breaths/min), peak inspiratory pressure (PIP, cmH_2O), positive end-expiratory pressure (PEEP, cmH_2O), and flow adjustment setting. PIP is reached in every test condition except for case 2, which is approximately 2.4 cmH_2O below the set point.

Fittings and 3D Printed Adapters

Standardized fittings were selected whenever possible to ease part sourcing in the event that engineers replicating the system need to swap out a component, possibly as the result of sourcing constraints within their local geographic area. Many fittings are American national pipe thread (NPT) standard, or conform to the respiratory circuit tubing standards (15 mm I.D./22 mm O.D.). To reduce system complexity and sourcing requirements of specialized adapters, a number of connectors, brackets, and manifold are provided as 3D printable parts. All 3D printed components were print-tested on multiple 3D printers, including consumer-level devices produced by MakerBot, Prusa, FlashForge, and Creality3D.

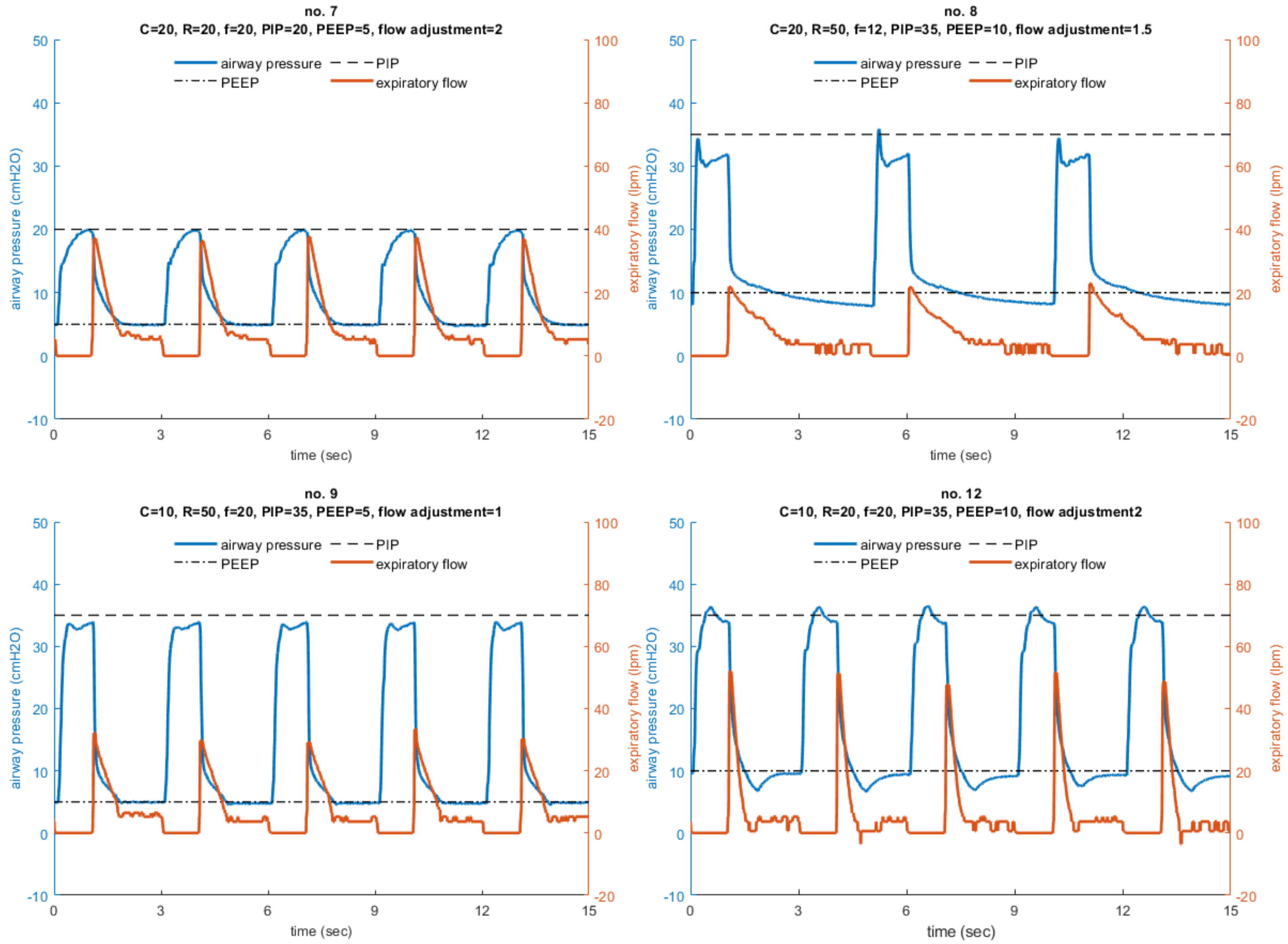

Fig 2. Performance results of the ISO 80601-2-80:2018 pressure controlled ventilator standard tests with an intended delivered tidal volume of 300 mL. For each configuration the following parameters are listed: the test number (from table 201.105 in the ISO standard), the compliance (C, mL/cmH_2O), linear resistance (R, cmH_2O/L/s), respiratory frequency (breaths/min), peak inspiratory pressure (PIP, cmH_2O), positive end-expiratory pressure (PEEP, cmH_2O), and flow adjustment setting. PIP is reached in every test condition.

Pressure Regulator

The fixed pressure regulator near the inlet of the system functions to step down the pressure supplied to the proportional valve to a safe and consistent set level of 50 psi. It is essential to preventing the over-pressurization of the system in the event of a pressure spike, eases the real-time control task, and ensures that downstream valves are operating within the acceptable range of flow conditions.

Proportional Valve

The proportional valve is the first of two actuated components in the system. It enables regulation of the gas flow to the patient via the PID control framework, described in a

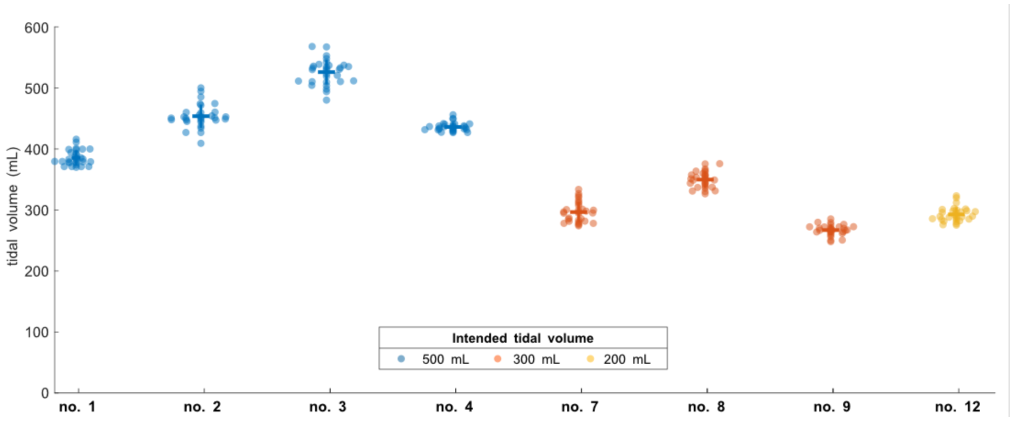

Fig 3. VTE estimates. Tidal volume performance for the ISO 80601-2-80:2018 pressure controlled ventilator standard tests, averaged across 30 breath cycles for each condition.

following section. A proportional valve upstream of the respiratory circuit enables the controller to modify the inspiratory time, and does not present wear limitations like pinch-valves and other analogous flow-control devices. The normally closed configuration was selected to prevent over-pressurization of the lungs in the event of system failure. Details are provided in the next section.

Sensors

The system includes an oxygen sensor for monitoring oxygen concentration of the blended gas supplied to the patient, a pressure sensor located proximally to the patient mouth along the respiratory circuit, and a spirometer, consisting of a plastic housing (D-Lite, GE Healthcare) with an attached differential pressure sensor, to measure flow. Individual sensor selection will be described in more detail in a following section. The oxygen sensor read-out is used to adjust the manual gas blender and to trigger alarm states in the event of deviations from a setpoint. The proximal location of the primary pressure sensor was selected due to the choice of a pressure-based control strategy, specifically to ensure the most accurate pressure readings with respect to the patient's lungs. Flow estimates from the single expiratory flow sensor are not directly used in the pressure-based control scheme, but enable the device to trigger appropriate alarm states in order to avoid deviations from the tidal volume of gas leaving the lungs during expiration. The device does not currently monitor gas temperature and humidity due to the use of an HME rather than a heated humidification system.

Pressure Relief Valve

A critical safety component is the pressure relief valve (alternatively called the "pressure release valve", or "pressure safety valve"). The proportional valve is controlled to ensure that the pressure of the gas supplied to the patient never rises above a set maximum level. The relief valve acts as a backup safety mechanism and opens if the pressure exceeds a safe level, thereby dumping excess gas to atmosphere. Thus, the relief valve in this system is located between the proportional valve and the patient respiratory circuit. The pressure relief valve we source cracks at 1 psi ($\sim$70 cmH_2O).

Anti-Suffocation Check Valve

A standard ventilator check valve (alternatively called a "one-way valve") is used as a secondary safety component in-line between the proportional valve and the patient respiratory circuit. The check valve is oriented such that air can be pulled into the system in the event of system failure, but that air cannot flow outward through the valve. A standard respiratory circuit check valve is used because it is a low-cost, readily sourced device with low cracking pressure and sufficiently high valve flow coefficient (Cv).

Bacterial Filters

A medical-grade electrostatic filter is placed on either end of the respiratory circuit. These function as protection against contamination of device internals and surroundings by pathogens and reduces the probability of the patient developing a hospital-acquired infection. The electrostatic filter presents low resistance to flow in the airway.

Standard Respiratory Circuit

The breathing circuit which connects the patient to the device is a standard respiratory circuit: the flexible, corrugated plastic tubing used in commercial ICU ventilators. Because this system assumes the use of an HME to maintain humidity levels of gas supplied to the patient, specialized heated tubing is not required.

Heat and Moisture Exchanger (HME)

A Heat and Moisture Exchanger is placed proximal to the patient. This is used to passively humidify and warm air inspired by the patient. HMEs are the standard solution in the absence of a heated humidifier. While we evaluated the use of an HME/F which integrates a bacteriological/viral filter, use of an HME/F increased flow resistance and compromised pressure control.

Pressure Sampling Filter

Proximal airway pressure is sampled at a pressure port near the wye adapter, and measured by a pressure sensor on the sensor PCB. To protect the sensor and internals of the ventilator, an additional 0.2 µm bacterial/viral filter is placed in-line between the proximal airway sampling port and the pressure sensor. This is also a standard approach in many commercial ventilators.

Expiratory Solenoid

The expiratory solenoid is the second of two actuated components in the system. When this valve is open, air bypasses the lungs, thereby enabling the lungs to de-pressurize upon expiration. When the valve is closed, the lungs may inflate or hold a fixed pressure, according to the control applied to the proportional valve. The expiratory flow control components must be selected to have a sufficiently high valve flow coefficient (Cv) to prevent obstruction upon expiration. This valve is also selected to be normally open, to enable the patient to expire in the event of system failure.

Manual PEEP Valve

The PEEP valve is a component which maintains the positive end-expiratory pressure (PEEP) of the system above atmospheric pressure to promote gas exchange to the lungs.

A typical COTS PEEP valve is a spring-based relief valve which exhausts when pressure within the airway exceeds a fixed limit. This limit is manually adjusted via compression of the spring. Various low-cost alternatives to a COTS mechanical PEEP valve exist, including the use of a simple water column, in the event that PEEP valves become challenging to source. We additionally provide a 3D printable PEEP valve alternative which utilizes a thin membrane, rather than a spring, to maintain PEEP.

2.2 Actuator Selection

When planning actuator selection, it was necessary to consider the placement of the valves within the larger system. Initially, we anticipated sourcing a proportional valve to operate at very low pressures (0-50 cmH_2O) and sufficiently high flow (over 120 LPM) of gas within the airway. However, a low-pressure, high-flow regime proportional valve is far more expensive than a proportional valve which operates within high-pressure ($\approx$ 50 psi), high-flow regimes. Thus, we designed the device such that the proportional valve would admit gas within the high-pressure regime and regulate air flow to the patient from the inspiratory airway limb.

Conceivably, it is possible to control the air flow to the patient with the proportional valve alone. However, we couple this actuator with a solenoid and PEEP valve to ensure robust control during PIP (peak inspiratory pressure) and PEEP hold, and to minimize the loss of O2-blended gas to the atmosphere, particularly during PIP hold.

Proportional Valve Sourcing

Despite designing the system such that the proportional valve could be sourced for operation within a normal inlet pressure regime ($\approx$ 50 psi), it was necessary to search for a valve with a high enough valve flow coefficient (Cv) to admit sufficient gas to the patient. We sourced an SMC Corporation PVQ31-5G-23-01N valve with stainless steel body in the normally-closed configuration. This valve has a port size of 1/8" (Rc) and has previously been used in respiratory applications. Although the manufacturer does not supply Cv estimates, we empirically determined that this valve is able to flow sufficiently for the application.

Expiratory Solenoid Sourcing

When sourcing the expiratory solenoid, it was necessary to choose a device with a sufficiently high valve flow coefficient (Cv) which could still actuate quickly enough to enable robust control of the gas flow. A reduced Cv in this portion of the circuit would restrict the ability of the patient to exhale. Initially, a number of control valves were sourced for their rapid switching speeds and empirically tested, as Cv estimates are often not provided by valve manufacturers. Ultimately, however, we selected a process valve in lieu of a control valve to ensure the device would flow sufficiently well, and the choice of valve did not present problems when implementing the control strategy. The SMC Corporation VXZ250HGB solenoid valve in the normally-open configuration was selected. The valve in particular was sourced partially due to its large port size (3/4" NPT). If an analogous solenoid with rapid switching speed and large Cv cannot be sourced, engineers replicating our device may consider the use of pneumatically actuated valves driven from air routed from a take-off downstream of the pressure regulator.

Manual PEEP Valve Sourcing

The PEEP valve is one of the few medical-specific COTS components in the device. The system configuration assumes the use of any ventilator-specific PEEP valve

(Teleflex, CareFusion, etc.) coupled with an adapter to the standard 22 mm respiratory circuit tubing. In anticipation of potential supply chain limitations, as noted previously, we additionally provide the CAD models of a 3D printable PEEP valve.

2.3 Sensor Selection

We selected a minimal set of sensors with analog outputs to keep the system design sufficiently adaptable. If there were a part shortage for a specific pressure sensor, for example, any readily available pressure sensor with an analog output could be substituted into the system following a simple adjustment in calibration in the controller. Our system uses three sensors: an oxygen sensor, an airway pressure sensor, and a flow sensor with availability for a fourth addition, all interfaced with the Raspberry Pi via a 4-channel ADC (Adafruit ADS1115) through an I^2C connection.

Oxygen Sensor

We selected an electrochemical oxygen sensor (Sensironics SS-12A) designed for the range of FiO2 used for standard ventilation and in other medical devices. The cell is self-powered, generating a small DC voltage (13-16 mV) that is linearly proportional to oxygen concentration. The output signal is amplified by an instrumentation amplifier interfacing the sensor with the Raspberry Pi controller (see Electronics Design section). This sensor is a wear part with a lifespan of $\approx$ 6 years under operation at ambient air; therefore under continuous ventilator operation with oxygen-enriched gas, it will need to be replaced more frequently. This part can be replaced with any other medical O2 sensor provided calibration is performed given that these parts are typically sold as raw sensors, with a 3-pin molex interface. Moreover, the sensor we specify is compatible with a range of medical O2 sensors, including the Analytical Industries PSR-11-917-M or the Puritan Bennett 4-072214-00, so we anticipate abundant sourcing options. The linearity of this sensor is $\leq$ 2% and the repeatability is $\pm$1% volume O2 at 100% O2 applied for 5 minutes.

Pressure Sensor (Airway)

We selected a pressure sensor with a few key characteristics in mind: 1) the sensor had to be compatible with the 5V supply of the Raspberry Pi, 2) the sensor's input pressure range had conform to the range of pressures possible in our device (up to 70 cmH_2O, the pressure relief valve's cutoff), and 3) the sensor's response time had to be sufficiently fast. We selected the amplified middle pressure sensor from Amphenol (1 PSI-D-4V), which was readily available, with a measurement range up to 70 cmH_2O and an analog output voltage span of 4 V. Moreover, the decision to utilize an analog sensor is convenient for engineers replicating the design, as new analog sensors can be swapped in without extensive code and electronics modifications, as in the case of I^2C devices which require modifications to hardware addresses. Other pressure sensors from this Amphenol line can be used as replacements if necessary. The linearity of this sensor is $\pm$ 0.5% f.s., with 1% span shift across 5°C to 50°C.

Spirometer

Because flow measurement is essential for measuring tidal volume during pressure-controlled ventilation, medical flow sensor availability was extremely limited during the early stages of the 2020 COVID-19 pandemic, and supply is still an issue. For that reason, we looked for inexpensive, more easily sourced spirometers to use in our system. We used the GE D-Lite spirometer, which is a mass-produced part and has

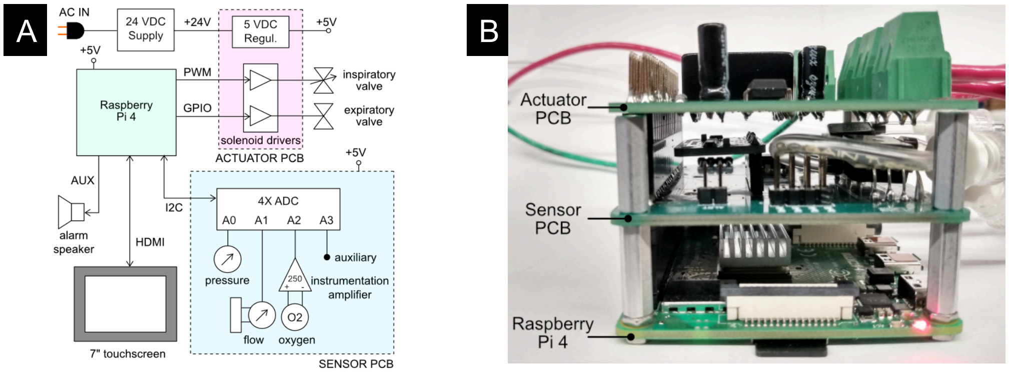

Fig 4. Overview of PVP1 electronics. A Block diagram. System power is supplied by AC through an uninterruptible power supply (UPS) regulated to 24 VDC by a switched-mode power supply, to be used to drive the valves. This DC is further regulated to 5 VDC to supply power to the Raspberry Pi and associated sensors. The sensor PCB coordinates the inputs of the pressure and oxygen sensors. The actuator PCB amplifies control signals from the Raspberry Pi to drive the valve solenoids. I/O is provided by a 7" touchscreen and an alarm speaker. **B** Photograph of the modular system electronics. Two modular PCB "hats" stack onto the Raspberry Pi via 40-pin stackable headers: the sensor PCB and the actuator PCB.

been used in hospitals for nearly 30 years. The D-Lite sensor is inserted in-line with the flow of gas on the expiratory limb, and two ports are used to measure the differential pressure drop resulting from flow through a narrow physical restriction. The third pressure-measurement port on the D-Lite is blocked by a male Luer cap, but this could be used as a backup pressure measurement port if desired. An Amphenol 5 INCH-D2-P4V-MINI was selected to measure the differential pressure across the two D-Lite takeoffs. As with the primary (absolute) pressure sensor, this sensor was selected to conform to the voltage range of the Raspberry Pi, operate within a small pressure range, and have a sufficiently fast response time (partially as a function of the analog-to-digital converter). Also, this analog sensor can be readily replaced with a similar analog sensor without substantial code/electronics modifications. The linearity of this sensor is ± 0.5% (maximum 0.25%) f.s., with 1% span shift across 5°C to 50°C.

2.4 Electronics Design

The components of PVP1 are coordinated by a Raspberry Pi 4 board, which runs the graphical user interface, administers the alarm system, monitors sensor values, and sends actuation commands to the valves (Fig 4). We elected to use a single Raspberry Pi rather than a computer connected to a dedicated microcontroller to minimize design complexity and to unify PVP1's software in flexible, extensible, high-level Python modules. Dedicated microcontrollers are typically used because they run a single program without interruption, but they typically require ventilation control logic to be written in low-level C, and add an additional point of failure and inflexibility in the communication API between the computer and microcontroller. By taking advantage of the Raspberry Pi's multiple processing cores and designing interfaces to a low-level hardware control daemon, PVP1 is capable of controlling ventilation at well below the latency of its hardware.

The main power to the systems is supplied by a DIN rail-mounted 150W 24V supply, which drives the inspiratory valve (4W) and expiratory valves (13W). This voltage is converted to 5V by a switched mode PCB-mounted regulator to power both the

Table 1. Actuator PCB bill of materials.

Ref	Part	Purpose
J2	2-pin screw terminal; 5.08 mm pitch; PCB mount	Connects to 24 V supply
J3	2-pin screw terminal; 5.08 mm pitch; PCB mount	Connects to on/off expiratory valve
J4	2-pin screw terminal; 5.08 mm pitch; PCB mount	Connects to inspiratory valve; driven by PWM
J5	40-pin stackable RPi header	Connects board to RPi
J6	2-pin 2.54 mm header	Jumper between 5V and Raspberry Pi
C1	100 µF; 16 V	5 V rail filter cap
C2	6.8 µF; 50 V	24 V rail filter cap
C3	6.8 µF; 50 V	24 V rail filter cap
U1	ULN2003A	Darlington BJT array to drive solenoids
U2	CUI PDQ15-Q24-S5-D	24 to 5 V DC–DC converter

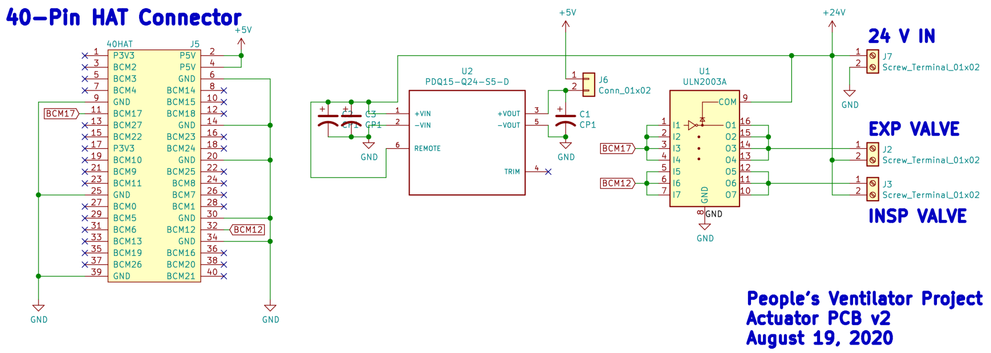

Fig 5. Schematic of the actuator printed circuit board. The actuator board serves two purposes: provide a 5V power rail, and provide an interface between the Raspberry Pi and the valves using a set of transistor arrays as solenoid drivers.

Raspberry Pi and sensors. This power is transmitted across the PCBs through the stacked headers when required.

The core electrical system consists of two modular board "hats", a sensor board and an actuator board, that stack onto the Raspberry Pi via 40-pin stackable headers (Fig 4B). The modularity of this system enables individual boards to be revised or modified to adapt to component substitutions if required.

Actuator Board

The purpose of the actuator board is twofold:

1. Regulate the 24V power supply to 5V (using a CUI Inc PDQE15-Q24-S5-D DC-DC converter)

2. Interface the Raspberry Pi with the inspiratory and expiratory valves through an array of solenoid drivers (ULN2003A Darlington transistor array)

The specific layout of the printed circuit board is available on the project web page. In Fig 5, we show a circuit schematic. Provided below in Table 1 is a detailed bill of materials.

Sensor Board

The sensor board interfaces four analog output sensors with the Raspberry Pi via I^2C commands to a 12-bit 4-channel ADC (Adafruit ADS1015). We have left open a single

Table 2. Sensor PCB bill of materials.

Ref	Part	Purpose
J1	40-pin stackable RPi header	Connects board to the Raspberry Pi
J2	4-pin 2.54 mm header	I^2C connector if desired
J3	2-pin 2.54 mm header	Connects ALRT pin from ADS1115 to RPi if needed
J4	3-pin 2.54 mm header or 3 pin fan extension cable	Connects board to oxygen sensor
R1	330 Ohm resistor	Sets gain for INA126
C1	10 µF; 25 V	Cap for TL7660
C2	10 µF; 25 V	Cap for TL7660
U1	TL7660; DIP8	Rail splitter for INA126
U2	INA126; DIP8	Instrumentation amplifier for oxygen sensor output
U3	Amphenol 5 INCH-D2-P4V-MINI	Differential pressure sensor (for flow measurement)
U4	Adafruit ADS1115	4x 12-bit ADC
U5	Amphenol 1 PSI-D-4V-MINI	Airway pressure sensor
U6		Auxiliary analog output sensor slot

sensor port for future use. To include more sensors, a mild modifications of these circuits will be necessary. The specific arrangement of sensors featured in PVP1 is as follows.

1. An airway pressure sensor (Amphenol 1 PSI-D-4V-MINI).
2. A differential pressure sensor (Amphenol 5 INCH-D2-P4V-MINI) to report the expiratory flow rate through a D-Lite spirometer.
3. An oxygen sensor (Sensiron SS-12A) whose 13 mV differential output signal is amplified 250-fold by an instrumentation amplifier (Texas Instruments INA126).
4. A fourth auxiliary slot for an additional analog sensor (unused).

A set of additional header pins allows for digital sensors (such as the Sensiron SFM3300 flow sensor) to be interfaced with the Raspberry Pi directly if desired. Fig 6 contains a schematic drawing of the circuit, and Table 2 its bill of materials. As with the actuator board, PCB layout and gerber files are available on the project web page.

3 Software Design

The software was modularly designed to facilitate future adaptation to new hardware configurations and ventilation modes. We carefully designed APIs for each of the modules to a) make them easily inspected and configured and b) make it clear to future developers how to adapt the system to their needs. The software has complete API-level documentation, making it so no part of the system is a black box.

All software development was done on GitHub, including a continuous-integration and automated testing suite with $> 99\%$ code coverage and thorough code reviews of the core routines. All code was tested in parallel with the automated testing suite and on the physical device itself. The source code is publicly available on our git repository, from which we also compile the documentation.

3.1 Software Architecture

The software is divided into two independent GUI and controller processes (Fig 7). The GUI process provides an interface to control and monitor ventilation, and the controller process handles the ventilation logic and interfaces with the hardware. Inter-process

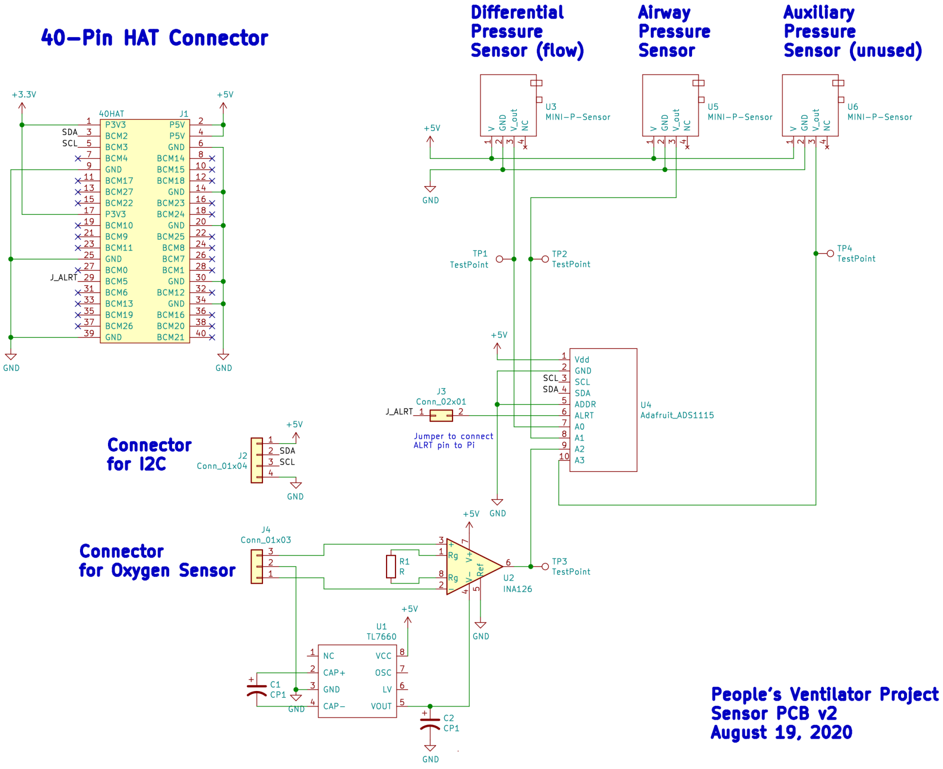

Fig 6. Schematic of the sensor printed circuit board. The sensor board provides an interface to four analog sensors using a 12-bit 4-channel analog-digital-converter (an Adafruit ADS1015) that communicates with the Raspberry Pi via I^2C.

communication is mediated by a coordinator module via xml-rpc. Several common modules facilitate system configuration and constitute the inter-process API. We designed the API around a unified, configurable `values` module that allow the GUI and controller to be reconfigured while also ensuring system robustness and simplicity.

The multiprocess model has several key advantages over the single-process model:

1. **Robustness:** Since the GUI and controller are independent, a failing process can be restarted without interrupting the other.

2. **Efficiency:** Both processes can operate on their own processor core, allowing the controller to do rapid control operations without being interrupted by slower GUI operations.

3. **Flexibility:** Because the rapid controller process is separate from the slower GUI process, our system uses a single low-cost computer rather than a computer and an additional dedicated microprocessor. By using a single computer we were able to write the entire program in a single, high-level programming language and avoid the complexity induced by needing to design a separate controller firmware in low-level C. As such, it is easier to develop additional controller modules, GUI widgets, and hardware interfaces to implement different ventilation modes and hardware configurations in the future.

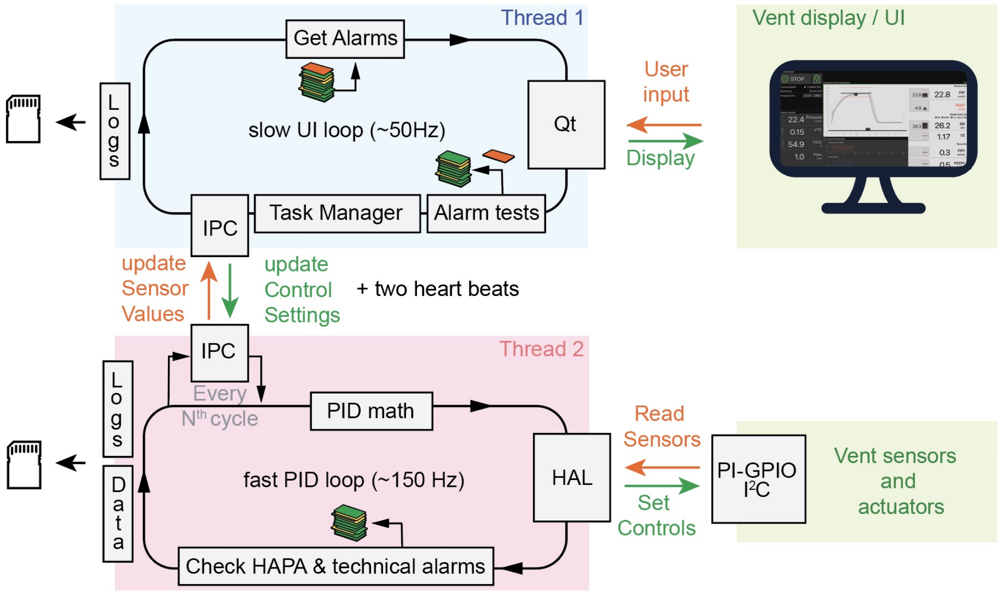

Fig 7. Overview of the software architecture. The user interface (UI) is controlled by a UI thread, that communicates with a second, faster, thread that implements the PID logic, by xml-rpc. The control loop is connected to hardware via the hardware abstraction layer (HAL). Both threads check for alarms; lower priority in the UI thread, and high priority in the controller thread, like HAPA (high airway pressure alert). The stack of cards illustrates a shared alarm-manager, which intelligently manages alarms, by keeping track of the various alarm types, and sorting by importance. The individual processes add to this stack.

General Software Considerations

Across all software modules, we made sure to have a well-defined startup/stop sequence, to store all relevant raw data and measure the aging of components:

PVP1 should only be started, and stopped, using the provided user interface. Hard termination of the code, i.e. without allowing any more CPU time for executing commands, might freeze the valves in position, conceivably damaging the lung. It is therefore imperative to set valves into a safe positions, specifically, closing the inspiratory valve, to open the expiratory valve and thereby to relieve the system of any residual pressure and protect against future pressure-buildup. The same code has to be executed if any `Exception` occurs.

PVP1 continuously logs raw data into compressed hdf5 files. Pressure, the key variable of the controller, is sampled and stored at the speed of the controller main loop, while flow is only monitored during exhalation when the expiratory valve is open, and oxygen-concentration is measured every five seconds (see controller). In addition, we keep a log of events (such as alarms and derived quantities) and continuously produce human-readable logs as plain text that log overall program state and progress.

As hardware components age (e.g. the oxygen sensor and the valves), it is critical for the software to measure the time when it was first activated, and not just the number of breath cycles performed. To this end, the software stores a set of varbiables upon first activation, in the file `prefs.json` including the time of first start.

Core-load of the entire software package was $\approx 60\%$, and $\approx 40\%$ for the two processes, and $\approx 15\%$ for pigpiod, the demon that performs communication with the periphery. PVP1 thus puts the Raspberry Pi's ARM Cortex-A72 CPU with its four

processor cores

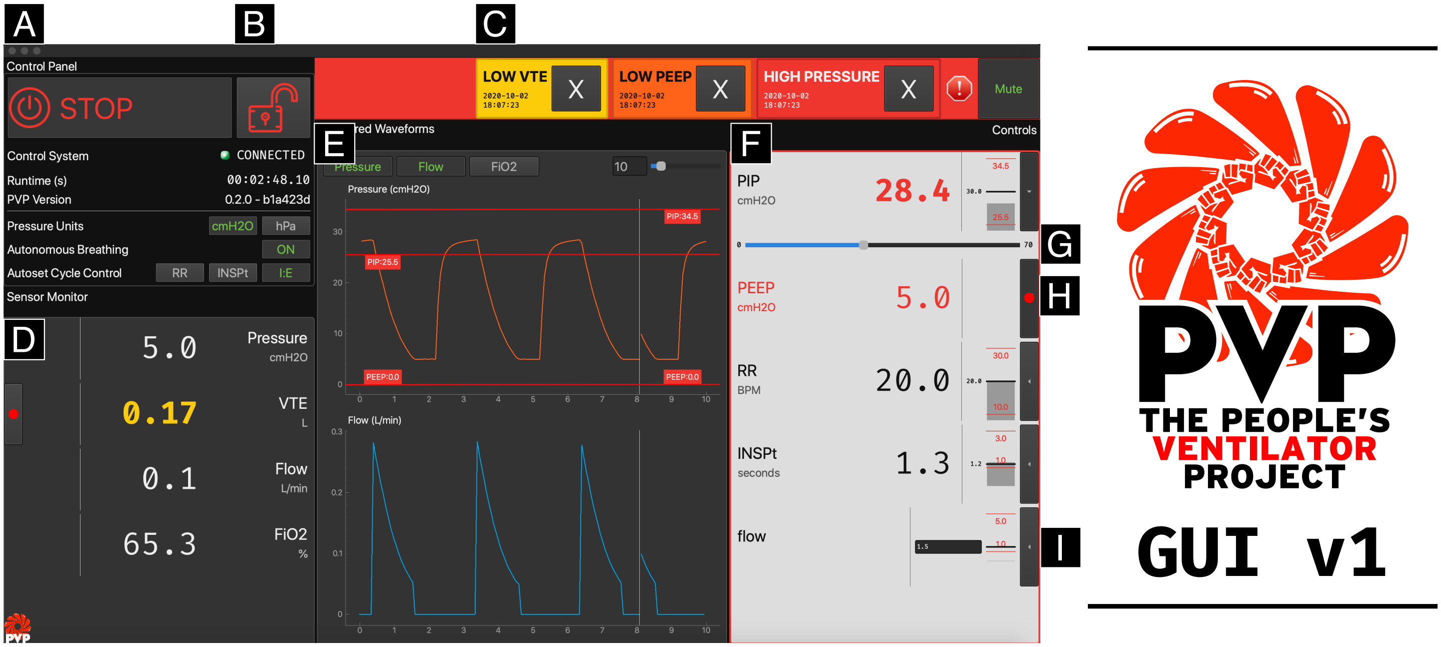

Fig 8. PVP1 GUI Design: The PVP1 GUI is composed of modular components to provide a uniform interaction syntax but also allow reconfiguration for different ventilation modes and hardware configurations. The GUI is broadly segmented into three columns: system monitoring components on the left (**A, D**), waveform plots of sensor values in the center (**E**), and ventilation controls on the right (**F**). A control panel (**A**) controls and displays basic system operating status. To prevent accidental changes, controls are locked (**B**) by default (but are unlocked in this figure). Beneath the control panel, a series of widgets in a "Monitor" column (**D**) display the values of sensors that are not used to control ventilation. The same widgets are to populate the rightmost "Control" column (**F**) to set the operating parameters of the ventilator. Ventilator control settings can be input with the touchscreen or mouse (with a slider, **G**), with the keyboard (in a text-entry box, **I**), or by recording recent sensor values (with a "record" button, **H**). By using a unified system of components across the whole interface, we can accomodate diverse hardware configurations (e.g. rather than setting a computer-controlled `PEEP` in the interface, we use a mechanically controlled `PEEP` valve and set alarm thresholds by recording sensed values) and alarm conditions (`VTE` is a derived sensor value rather than a control value, but needs to have patient-calibrated alarm thresholds) while maintaining a consistent interaction syntax. Alarm thresholds are automatically calculated as some multiple of the set value (e.g. a HAPA alarm is 115% of set `PIP`). Alarm thresholds are represented numerically and graphically in both the relevant control widget and waveform plot (red horizontal lines in **E** and **F**). Alarms are displayed as "Alarm Cards" (**C**) color coded and ordered by alarm severity. Color codes are also reflected in the relevant displayed parameter (e.g. yellow displayed `VTE` value in **D** to match the low-severity `LOW VTE` alarm in **C**), allowing clinicians to quickly identify and attend to the cause of an alarm. Screenshot of actual GUI running PVP's built in stimulation mode.

under only a mild load. Memory load was about 20% or 200 MB. The Raspberry Pi allows CPU frequency scaling, which enables the operating system to scale the CPU frequency up or down depending on demand. The governor, which regulates this scaling, was set to performance to deactivate this feature. In addition, stand-by and screensaver were deactivated.

GUI Design

The GUI was designed to semantically segment system control, ventilation control, sensor monitoring, and alarm status while maintaining a unified syntax of interaction (Fig 8). We attempted to design the GUI so that it was intuitive enough to use without documentation by clearly separating and labeling the sections of the interface, providing

few, clear points of control and interaction, and giving the user informative prompts to guide their use.

The GUI makes global system status clearly intelligible from across the room by using a uniform language of "alarm cards" (Fig 8C) and color codes that indicate the severity and source of alarms to allow medical professionals to identify and address them as soon as possible. Alarm limits are displayed graphically next to the control widget and overlaid on relevant waveform plots (Fig 8E, F) so values approaching a limit are clearly visible in either modality. The alarm system and sound design are described further in the section 3.3 below.

Ventilation controls and sensor monitors (Fig 8D, F) are displayed using a single widget class which makes them visually distinct but behave identically when setting alarm limits, displaying values, etc. These value display widgets support setting ventilation controls using the mouse/touchcreen (with a slider), keyboard, or by recording and averaging recent sensor values (Fig 8G, H, I). All components of the GUI are modular and generated programmatically from the shared `values` module, which allows monitored and controlled values to be trivially reconfigured for different hardware configurations and ventilation modes while keeping a consistent API between the GUI and controller.

3.2 Supported Alarms

We support the following EUA alarms:

- Low Airway Pressure Alarm (LAPA) if target pressures (PIP or PEEP) is not reached, see Fig 8.
- High Airway Pressure Alarm (HAPA) if pressure exceeds a critical limit, see Fig 8.
- Hypoventilation Alarm if measured VTE is smaller than target, where target is defined in the first few breath cycles.
- Tidal Volume not met Alarm if VTE is too small or too large.
- PEEP Alarm, if PEEP is not reached, see Fig 8.
- Obstruction Alarm.
- Disconnect / high leakage Alarm.
- Oxygenation alarm, if the oxygen value deviates more than 5% from setpoint.
- Technical Alarm: A general class of alarms that are triggered whenever the software cannot work reliably.

3.3 Alarm Design

Alarms are implemented as configurable `Alarm_Rules` coordinated by a centralized `Alarm_Manger`. An alarm rule describes a) the `Conditions` for triggering an alarm, and b) the behavior and appearance of the alarm on the UI (Fig 9). Alarm conditions are implemented as composable classes that can accommodate complex triggering logic while remaining clear and inspectable (Fig 9, Lines 5-23).

Alarm Display - We try to balance salience of high severity alarms while minimizing unnecessary cognitive overhead by representing them as `Alarm_Cards` within an `Alarm_Bar` (Top of Fig 8). When no alarm is present, the bar is invisible, but takes the color of the highest-priority active alarm to give an unambiguous global status indicator. Alarm cards ensure each type of alarm is only represented once, allow

individual control over dismissal/silencing of alarms, and visually triage lower-priority alarms by keeping them ordered by severity. The behavior of an alarm card is also determined by its alarm rule (See caption of Fig 9), so that critical alarms are not missed while transient, lower-severity alarms do not clutter the interface. The color-code of an alarm card is reflected in the widget that controls the relevant parameter (e.g. the yellow `VTE` value in Fig 8), allowing attending physicians to quickly determine the source of alarms and how to correct them.

Example Alarm Rule

```
1  Alarm_Rule(
2    name       = LOW_PRESSURE,
3    latch      = False,
4    persistent = False,
5    conditions = (
6    (
7    AlarmSeverity.LOW,
8      ValueCondition(
9        value_name = ValueName.PIP,
10       limit      = LAPA_THRESHOLD_1,
11       mode       = 'min')
12   ),
13   (
14   AlarmSeverity.MEDIUM,
15     ValueCondition(
16       value_name = ValueName.PIP,
17       limit      = LAPA_THRESHOLD_2,
18       mode       = 'min'
19     ) + \
20     CycleAlarmSeverityCondition(
21       alarm_type = LOW_PRESSURE,
22       severity   = AlarmSeverity.LOW,
23       n_cycles   = 2))
24 ))
```

Fig 9. Example Low Pressure Alarm Rule. An Alarm Rule defines the behavior of an alarm in the GUI and the conditions for raising the alarm. Alarms can be `latched` (**L3**), where they cannot be visually dismissed until the alarm condition terminates or `persistent` (**L4**), where they will remain displayed until the user manually dismisses them. These settings ensure attending physicians never miss critical alarms, but are not overwhelmed with transient, low-severity alarms. Complex sets of `Conditions` for raising alarms can be described while remaining human-readable. This alarm has two severities (highlighted pink): a `LOW` severity alarm (**L7-11**) is triggered when `PIP` falls below some `LAPA_THRESHOLD`, which is escalated to a `MEDIUM` severity alarm (**L14-23**) if `PIP` falls below another threshold *and* the `LOW` severity alarm has been active for 2 breath cycles. Note how multiple conditions can be added (**L19**, literally with `+`) together, which allows triggering conditions to depend on multiple values, the states of other alarms, time, etc. In practice, rather than static alarm limits with a single value, all `Condition` values are updated from control values with some transformation (e.g. this threshold could be kept at 15% below set `PIP`), but these dependencies have been omitted for brevity.

Alarm Sounds - We designed a set of alarm sounds (available in our repository) to

be informative alert attending physicians while avoiding alarm fatigue. Only a single alarm sound is played at a time, and the alarm sound reflects the highest severity active alarm and the duration it has been active.

Alarm sounds are short (≈ 300 ms) tone sequences, and severity of alarm is represented by pitch and the number of tones in each sequence - i.e. a low-severity alarm is a repeating single low tone, and a high-severity alarm is a repeating sequence that adds two higher tones. At alarm onset, alarm sounds are low-pass filtered and have a lengthened attack and decay to soften their presentation while the physician first begins attending to the alarm condition. As alarms remain on, the filter, attack, and decay of the tone smoothly decrease to transition the sound to sharper, more urgent "clicks".

All sounds use brief (≈ 40 ms), noncontinuous tones that are silent for at least half of their duty cycle, leaving space for conversation and other sound. We attempt to disambiguate our alarms from other auditory alarms that could be present in the room by underlaying a soft pneumatic "sucking" sound synchronized to the tone sequences. Alarm sounds follow the same persistence rules as the relevant alarm rule (Fig 9, caption), reducing alarm fatigue by allowing transient, low-priority alarms to automatically silence themselves. Alarm sounds can also be muted entirely, or by dismissing specific alarms ("Mute" and "X" buttons in alarm bar in Fig 8, respectively) so they function to inform clinicians about patient state and then get out of the way.

Controller Module

Control into a breathing cycle was accomplished with a hybrid system of state and PID control. During inspiration, we actively control pressure using a PID cycle to set the inspiratory valve. Expiration was then instantiated by closing the inpiratory, and opening the expiratory valve to passively release PIP pressure as fast as possible. After reaching PEEP, we opened the inspiratory valve slightly to sustain PEEP using the aforementioned manually operated PEEP-valve and to sustain a gentle flow of air through the system.

Active control during inspiration was constructed around a single PID-style control loop. Pressure values were measured proximal to the patient, and communicated to the Raspberry PI using I^2C. The Raspberry Pi compared this value against a user-provided target, and from that estimated proportional, derivative, and integral errors. Control coefficients were optimized manually on physical hardware by tuning against the set of EUA test conditions. The control-signal was then sent to the inspiratory and expiratory valves which caused a change in air-flow within ≈ 40 ms (related to the inductive load of the valve), while we ensure that the primary control loop ran at considerably higher speeds than this delay (c.f. Figs 10 and 12).

We also allow the user to adjust flow through the system, by controlling `PIP_TIME` which is a multiplier for the PID coefficients. For lungs with higher compliance, more air is required to inflate to comparable pressures. As this assessment requires experience, we left the control of maximum flow rate to the clinician. During the primary control loop, pressure values have to be read as fast as possible. As the principal bottleneck is the communication with hardware, we chose to only read pressure values during inspiration. Flow out of the lung and oxygen concentrations are therefore only measured during expiration, where pressure and flow-readings alternate, and a single oxygen reading is obtained every five seconds.

In addition to pressure control, our software continuously monitors for autonomous breaths, high airway pressure, and general system status. Autonomous breathing was detected by transient pressure drops below PEEP. A detected breath triggered a new breath cycle. High airway pressure is defined as exceeding a threshold pressure for a minimum time (as to not be triggered by a cough). This triggers an alarm, and an immediate release of air to drop pressure to PEEP. The Controller also assesses whether

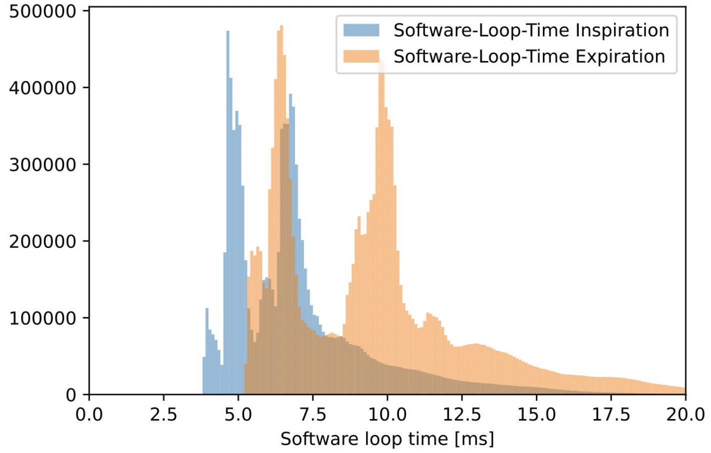

Fig 10. Histograms of the software loop time. Measurements of the software loop-time during inspiration (blue) and expiration (orange) over ca. 62h of breathing. Median inspiration loop time is 6.6 ms, median expiration loop time is 9.5 ms. Notice multiple peaks, depending on the addition of sensor readings, every one of which contributes $\approx$ 2.5 ms. The mechanical valves are a factor of $\approx$ 5 slower than the software .

numerical values are reasonable and updating over time (to test that the sensors did not get stuck). If this is not the case, it raises an technical alarm. All alarms are collected and maintained by an intelligent alarm manager, that provides the UI with the alarms to display in order of their importance. Note that such smart designs, while not universally adopted, will be standard in the next generation of mechanical ventilators [2].

Measuring the expiratory flow, $F(t)$ is sufficient to estimate VTE. To this end, we first estimated a baseline flow through the system, but not the lung (i.e. the flow to sustain PEEP, see section above). This was done by calculating a histogram of values in $F(t)$ during expiration in a moving window, and applying a rank-filter to these numbers to define the baseline flow $F_0(t)$. We then integrated the difference over the expiration window T to obtain

$$\mathrm{VTE} = \int_0^{\mathrm{T}} \mathrm{d}t\, F(t) - F_0(t).$$

Hardware I/O

The low-level firmware code is designed to be modular and makes extensive use of inheritance such that generic classes can be re-used for different applications, e.g. switching out a sensor, valve or ADC. In many cases, adapting the code to accommodate a new device requires re-writing a few lines of code.

We implement a hardware abstraction layer (HAL) to simplify the interaction between the controller and the firmware code so that all the complexity of the firmware code is hidden from the engineers who are developing the controls. Additionally, reconfiguration of the hardware is accomplished easily from the perspective of higher-level users due to the use of hardware config files.

For all communications over the GPIO pins of the Raspberry Pi, including sensor/ADC readings and valve control, we utilize the pigpio daemon [1]. The pigpiod is a standalone interface which runs in its own process and is written in C. It is significantly faster than the default GPIO libraries and provides additional features like hardware-generated PWM signals. The pigpio Python library provides Python bindings for inter-process communication with the pigpio daemon.

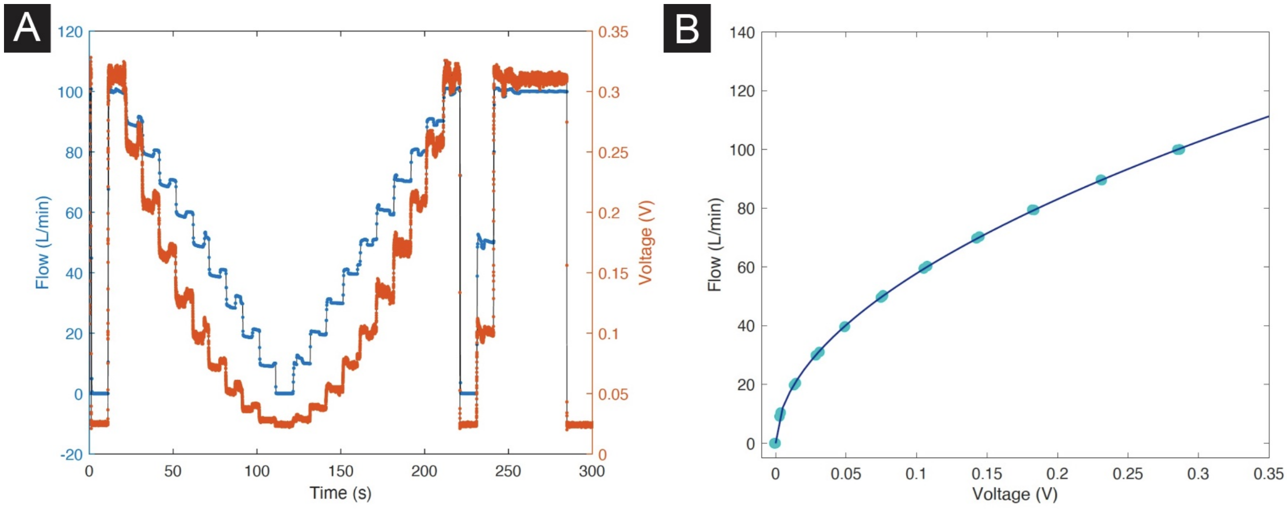

Fig 11. Calibration of the Flow Sensor. A Flow introduced in steps of 10 L/min ranging from 10 L/min to 100 L/min. Blue points represent the flow measured by the mass flow controller and orange points represent the voltage recorded from the Dlite sensor. **B** Obtained calibration Curve for the Flow Sensor. Measurements of the flow as a function of the voltage recorded from the sensor. Turquoise points are the averaged data from the calibration and the blue line is the fit $V = 192.6\, F^{1/1.91}$, where V is the flow in L/min and F is the sensor voltage.

Software speed

Following the implementation of the previously described main elements of the software, we performed an experiment to measure the speed of the primary control loop. More specifically, we saved the execution times of the primary loop while PVP1 was ventilating a lung simulator. The results are shown in Fig 10. Note the difference between inspiration and expiration; expiration is considerably slower, as additional sensor-reads for flow and oxygen sensing are required (see Hardware I/O section above). The Raspberry Pi allowed for the primary control loop to run with a median loop-time of the entire software package of 6.6 ms during inspiration and 9.5 ms during expiration.

4 Calibration and System Limitations

4.1 Calibration of the Flow Sensor

We calibrated the flow sensor using a mass flow controller (Alicat Scientific, MCR-100SLPM-D). The flow into the sensor was ramped up and then down in step of constant flow ranging from 10 L/min to 100 L/min (Fig 11A blue).

At each step the flow was imposed for 10s and the voltage from the sensor was recorded (Fig 11 orange). Data points up to 5s after each jump and 1s before the next jump were discarded. For the remaining 4s the voltage data points were averaged to match the 10 Hz recording frequency of the mass flow controller. Subsequently both the flow and the voltage data was averaged at each step and used to calculate the calibration curve in Fig 11B. To find the calibration curve the data was fit to a function of the form $V = a \cdot F^{1/k}$ where V is the flow in L/min and F is the sensor voltage. The parameters were determined to be $a = 192.6$ and $k = 1.91$.

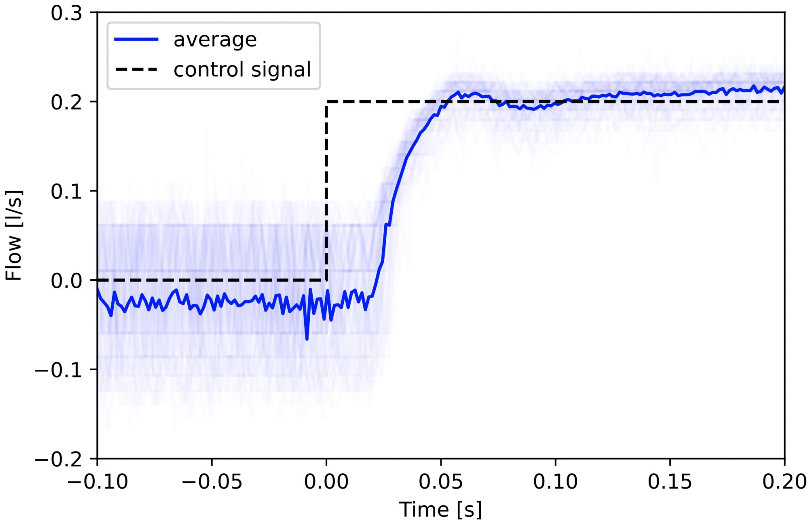

Fig 12. Measurements of the delay caused by the inspiratory valve. At time zero, the control signal to open the valve was sent and we monitored flow through the system. The thin blue lines illustrate 100 consecutive individual trials, the drawn blue line is the average, and the dashed line illustrates the control signal. 50% of the maximum flow was reached after 32 ms, 90% were reached after 53 ms.

4.2 Hardware Delay Characterization

A principle physical limitation of PVP1 is the physical process of controlling the valves. As mechanical devices with an inductive load, we expect a delay between the control signal, and an increase in flow. To make sure that this delay is the principle bottleneck of PVP1's contol, and not the limited speed of the Raspberry Pi, we aimed to perform such a measurement. To measure the delay of the inspiratory valve, we sent 100 consecutive 'open' and 'close' -commands to the inspiratory valve, while monitoring flow through the respiratory circuit.

Upon sending the control signal, flow increases with a delay of ≈ 50 ms. This is shown in Fig 12. We attribute this delay to the inductive load of the motor, and the finite time required for the mechanical opening of the valve. Note also, that this delay is considerably longer than the software-loop-time, and thus constitutes the principle bottleneck of the control system. This measurement was done with mild adjustments to the PVP1 software.

References

1. Instructions are available at http://abyz.me.uk/rpi/pigpio/ 18

2. Kacmarek RM (2011) The Mechanical Ventilator: Past, Present, and Future. Respiratory Care 56(8): 1170-1180. doi:10.4187/respcare.01420 18
