# Supplementary material for: PVP1—The People’s Ventilator Project: A fully open, low-cost, pressure-controlled ventilator research platform compatible with adult and pediatric uses
Source: PLoS One. 2022 May 11;17(5):e0266810. doi: 10.1371/journal.pone.0266810 (PMC9094548; doi:10.1371/journal.pone.0266810)
Supplement: Attachment — Submitted filename: Response to Reviewers.pdf [file pone.0266810.s003.pdf]

To whom it may concern,

We would like to express our gratitude to the editor and the reviewers for the efficient review process, for taking the time to read this manuscript and the associated data/materials, and their thoughtful and important comments. We believe that we could address all concerns, and that, in this process, the revised manuscript has improved considerably.

Reviewer #1: This paper presents the People's Ventilator Project (PVP1), a fully open-source and low cost ventilator which can serve as a research platform. While it has not been medically certified yet, it shows some promising results with artificial lungs in both adult and pediatric cases.

Overall, the paper is well written and provides an important addition to the literature. They authors make a strong case for the need for open source ventilators, while also highlighting that numerous other open source ventilators had to shift away from being open source due to external (primarily commercial and regulatory) pressures. The latter points to a need in societal changes to accommodate for open projects.

The authors can find my comments within the attached the PDF file. In addition to those, I'd like to mention that it is important that the images and tables move so that they are in a location that fits the flow of the paper. Right now, they really disrupt the reading experience. As a rule of thumb, it is good for figures and tables to follow after they have been introduced in the main body text. In conclusion, I am happy to recommend this paper for publication subject to the authors considering making the recommended minor changes.

We thank the reviewer for their kind words, and their positive assessment. We have accepted all suggested changes in the revised rendition (highlighted as blue). Furthermore, we have:

- Rearranged the figures, including the new Figure 5, to appear after their first occurrence in the text, or on the same page when possible.
- Provided an open know-how manifest to aid sharing the hardware design and documentation online. The file is contained in the github repository with pull request #149.
- Received an OSHWA certification, UID US002073.
- And made a snapshot of the repository available with https://doi.org/10.5281/zenodo.5933282

Reviewer #2: The manuscript presents an open hardware ventilator for educational and research purposes. The authors appear to have taken considerable care to ensure that the ventilator is also appropriate to pediatrics. Some thought has been taken to how the design may be deployed as a medical device but this aspect is some way off. The authors correctly identify this and they should be commended for being explicit and clear about the current status of their project.

We would like to thank the reviewer for the considerable amount of time and work that they put into their review. We believe that all of the points raised were fair, and important. They are addressed in the following paragraphs.

For context of this review my expertise is on open hardware design and transferring open hardware designs from within academia into distributed production in the global south. This review does not consider the ventilator performance itself. This aspect should be assessed by a reviewer with specific expertise.

We thank the reviewer for this disclosure. In the revised manuscript, we added a new paragraph "*Missing steps towards a medical device*" to more explicitly enumerate actions that need to be taken before pvp1 can be used as such. In this paragraph, we have also briefly sketched the EUA process, and brought up the ISO norms, mentioned below.

The manuscript regularly describes the project as "fully open", boasting that a "complete" web-portal is "optimized" for sharing the entire design, and that complete design decisions have been provided.

We wish to apologize for this. Our intention was not to show off. We have added a number of new citations to other published projects, changed these sentences mentioned above, and overall phrased the text more conservatively.

The software has indeed been developed using open source best practices. A github repository with almost 1,000 commits, and with numerous issues in the issue tracker, and with 73 descriptive merge requests. From this I am confident I could review and understand the software design. All software documentation is versioned in the same repository and rendered.

However, this is far from the case for the hardware. The assembly instructions are a Google document, the rendered bill of materials is truncated on the website making it impossible to read. The original CAD files needed to continue the design are provided. However, they all dumped haphazardly into a Google drive folder titled "Julie Magic Air Machine", complete with names such as "I_AM_AN_IDIOT_ADAPTER_15M_TO_22F.SLDPRT". There appears to have been no attempt to document the design history, nor to curate and archive the design somewhere more stable than a Google Drive.

I suggest that at the very least the team organise and curate the design files into a directory structure complete with README files in place to help someone navigate. Any design history that is available could be captured in CHANGELOG files, perhaps with older versions in sub directories. These files should then be archived somewhere such as Zenodo so that this manuscript can link to an unchanging identifier.

To clarify, I pick out the "idiot adapter" not to say that when we make mistakes they should be hidden, however without a structured history the context of what this means is lost. Mistakes are also recorded in the software history, but they are removed from the current version and identified clearly with descriptive commit messages.

Considering design decisions, what is provided is in the supplementary information. A few pages of top down design information do little to mitigate the vast quantities of design information that has been lost by not following the same excellent open practices that were followed for the software design. Of course the processes for collaborative open hardware design are far less established than they are for software.

We thank the reviewer for taking the time to go through the software and hardware documentation and the thoughtful feedback particularly on the hardware side of the project. We wholeheartedly agree with many of the concerns, and have addressed them as follows:

1. We agree that our existing hardware documentation should be supplemented. We have changed the hardware documentation to markdown files, maintained in the repository, and part of the online documentation. This is done with **Pull Request (PR) #148**
2. We followed the reviewer's advice and now provide the current hardware (with renamed parts, and including three earlier designs) in a directory contained in the git repository. This is done in **PR #149**. Earlier hardware designs are now contained in the `assets` subdirectory, together with a (manually compiled) changelog that documents major changes in the hardware.
3. In addition, we provide **several new paragraphs and an additional figure** in the discussion of the main manuscript, specifically section "*Design Process and Critical Iterations*", where we focus on PVP1's design history in the light of the two earlier versions. We refer to these versions as Mk1 and Mk2. For both of them, and the current iteration Mk3, we provide solidworks drawings, and part lists. This information became part of the repository with **PR #149**.
4. This PR also addresses readability of the bill-of-materials on the website, and in addition to a short preview on the website, provides a full CSV file with details.
5. **A new section in the discussion** narrates the hardware design changes to provide a reader with context to why the device is designed as is. This also contains our experiences of designing PVP1 during the COVID-19 pandemic.
6. As the reviewer points out, there are various reasons why hardware documentation does not reach the same level as software documentation. We have added a **new section** to the discussion, "*Challenges of Open Hardware*", in which we make these challenges explicit.
7. We also added a paragraph in "*Future Hardware Development Goals*", in which we invite readers to make pull requests, for example for changes of parts to metric.
8. In the revised section "*PVP1: An Open Source Project During a Pandemic*", we provide a history of how we developed a community, and how PVP1 aims to move forward.
9. We provide a DOI via Zenodo to a snapshot of the entire documentation and repository (at the time of resubmission) in the manuscript, that includes all the changes mentioned above: https://doi.org/10.5281/zenodo.5933282

The authors would do better to tone down the self-congratulation on how fully open they are and address head on the challenges they had in working openly on the hardware design. Comparing and contrasting these challenges to the standard practices that they mention for the software design would be both useful for future reference and would be more honest. The author should

look to the literature for other commentary or examples on the technical challenges of open hardware design, both generally and specifically in the case of open design of ventilators during the pandemic.

We thank the reviewer for stressing this point. The revised discussion contains (i) an additional paragraph where we discuss the difficulties of a genuine open hardware design, (ii) we have toned down the language of the manuscript and (iii) better describe the journey and history of the device during the covid epidemic.

Moving on..

The authors state that no specialist knowledge is required to reproduce the design. It should be clarified that this would not be true in the case of a medical device. It is worth explicitly clarifying that anyone looking to take the design to market would need to fully understand the device and take responsibility for it as a legal manufacturer.

We have made this important point more explicit in a dedicated paragraph in the "*Missing steps towards a medical device*" section of the discussion.

Of course this is why the design decisions mentioned above are so critical. The authors would do well to link the sharing of design decisions to medical device manufacturing in the context of international standards for medical device design such as ISO 13485 and ISO 14971. Due to the heavy software component IEC62304 should also be considered. I understand that the ventilator is not at the stage of a medical device, but putting the work needed to transition it to one in the context of the internationally harmonised standards is more useful than only considering only the FDA guidance (which is harmonised with international standards). The authors may find that documentation for the Global Harmonisation Task Force will help them put the work a more international context.

We refer to these efforts in the revised discussion in the same ""*Missing steps towards a medical device*" section.

Furthermore it is a shame that the ventilator has been designed using USA-only threads and fittings. My experience working in Africa is that when we have tried to replicate American designs that use USA-specific fixing, we need to significantly modify the design before it can be implemented. USA threads and fittings are available in limited capacity in Europe, but they require highly specialised suppliers. The rest of the world has standardized on metric, having spent years working in the USA I know that metric components are readily available from sources like McMaster-Carr. I suggest that the manuscript should temper its claims to have selected readily available components, or be specific that the components are only readily available in one single country.

We have made this explicit in the revised manuscript and aimed to make the design considerations explicit why, in an epidemic and us based in North America, these pipe fittings seemed like an appropriate choice. This is contained in the narrative paragraph on hardware history in the revised discussion, section "*Design Process and Critical Iterations*".

In conclusion the paper describes a ventilator designed during the pandemic. This ventilator has done more than many by ensuring that the original design files are available. However, the reality of the open hardware design does not meet the rose-tinted description in the manuscript, which largely implies (incorrectly) that best practices used for software were also followed for the ventilator. The manuscript and design are also highly USA focused. With curation and archiving of the hardware design, and significant tempering of the language, tone, and descriptions in the manuscript I believe this work should be published in PLOS ONE.

We thank the reviewer for these kind words, and hope that our resubmission can adequately address all their concerns.
